# Disrupting power hierarchies: applying a trauma- informed, intersectional, reflexive engagement strategy

**DOI:** 10.1186/s40900-026-00930-4

**Published:** 2026-07-01

**Authors:** Cara L. Brown, Patricia Thille, Rebecca Ganann, Teresa Allison, Simone Moorhouse, Gail Cathrine Pelletier, Patricia E. Rawsthorne, Aya El-Alawi

**Affiliations:** 1 Department of Occupational Therapy, College of Rehabilitation Sciences, Rady Faculty of Health Sciences, University of Manitoba, P304 - 770 Bannatyne Avenue, Winnipeg, MB R3E 0W2 Canada; 2 Department of Physical Therapy, College of Rehabilitation Sciences, Rady Faculty of Health Sciences, University of Manitoba, P304 - 770 Bannatyne Avenue, Winnipeg, MB R3E 0W2 Canada; 3 School of Nursing, Faculty of Health Sciences, McMaster University, 1280 Main St. W. HSC 3N25C, Hamilton, ON L8S 4K1 Canada; 4 Department of Occupational Therapy, College of Rehabilitation Sciences, Rady Faculty of Health Sciences, University of Manitoba, R106, 771 McDermot Ave, Winnipeg, Manitoba R3E 0T6 Canada; 5 College of Rehabilitation Sciences, Rady Faculty of Health Sciences, University of Manitoba, 771 McDermot Ave, Winnipeg, Manitoba R106 Canada; 6 Department of Occupational Therapy, College of Rehabilitation Sciences, Rady Faculty of Health Sciences, University of Manitoba, 771 McDermot Ave, Winnipeg, Manitoba R106 Canada

**Keywords:** Patient and public involvement, Patient research partners, Care transitions, Diversity, Equity, Inclusion, Engagement, Trauma-informed, Reflexivity, Power

## Abstract

People with lived experience in health care, like patients and their family and/or friend caregivers, are important to include in the research process to improve impact and relevance of research. However, these lived experience researchers (LERs) can be harmed if this engagement is done poorly, especially though tokenization, or through the reliving of health care related traumatic experience. Five LERs joined a research team that collaboratively developed a research proposal through seven workshops. The research topic was of care transitions from hospital to home, of which all five LERs had lived experience as patients or caregivers. To mitigate harm for the LERs, the project was designed with a trauma-informed, intersectional and reflexive approach, with a focus on disrupting historical power structures inherent in research and health care systems. The engagement design was planned a priori and uniquely incorporated regular feedback mechanisms and strategies to support reflexivity for all team members. This engagement strategy had some success in disrupting standard power hierarchies that persist in research teams and can impede meaningful LER engagement. Ultimately the LERs felt heard and understood by the team and developed strong relationships with the principal investigator (PI) and project coordinator, and they contrasted this experience with other health and research experiences where they perceived their input to have less weight. The reflexive approach revealed what aspects of the engagement were going well, and which continued to be plagued by historical power and hierarchies inherent in academic and health systems. While the continual feedback process did not allow the team to completely overcome these hierarchies, it brought them to light so that they could be addressed as much as possible within the context of the project, and we share three examples in this commentary. We hope that this detailed description of developing a research proposal using a power-focused framework with a team with multiple LERs helps to address the gap in the literature about how researchers can support the reduction of power hierarchies when engaging LERs.

**Supplementary information:**

The online version contains supplementary material available at 10.1186/s40900-026-00930-4.

## Abstract (summary)

People with lived experience in health care, like patients and their family and/or friend caregivers, are important to include in the research process as research team members. However, these lived experience researchers (LERs) can be harmed if this engagement is done poorly, especially when dealing with a topic where the LERs have had harmful experiences. This was the case when academic researchers wanted to include LERs on a research team to develop a project on care transitions from hospital to home for older adults. To mitigate harm for the LERs as much as possible, the project was designed with a trauma-informed, intersectional and reflexive approach. A trauma-informed approach promotes safety and choice to avoid re-triggering of past traumas. An intersectional approach acknowledges that multiple intersecting systems of oppression contribute to an individual or group’s power and privilege and works to disrupt these systems. Reflexivity is a personal process of examining and critiquing the assumptions and worldviews of oneself and one’s contexts to challenge the status quo and move towards equity and justice. The engagement design included feedback mechanisms to determine what was going well with the engagement strategy, and to make necessary adjustments. Through this paper, we aim to support researchers in planning how to conduct patient research engagement in a way that reduces power hierarchies and harm to LERs so that LERs can participate fully and safely in research.

**Supplementary information:**

The online version contains supplementary material available at 10.1186/s40900-026-00930-4.

## Introduction

Research to reduce adverse outcomes for hospital to home transition for older adults has been ongoing for decades [1]. Despite this, older adults continue to be at high risk for multiple harms during the transition process with damaging outcomes. Older adults are more likely to be discharged home with delirium or at risk of falls, to have an adverse event at home due to flawed hospital to community communication, and to have a readmission to hospital that could have been preventable with adequate services and supports [2, 3]. Finding solutions to this ongoing issue has been challenging due to the complexity of care transitions where coordination between multiple health care settings, professionals and organizations is required [4]. Care is easily disjointed and disconnected, otherwise known as fragmented, when it is delivered by multiple social and health care programs, each with unique cultural and organizational values and care approaches [4, 5]. Further, those at most risk are those with poor determinants of health, such as a limited social network and low income, which is more common in the older adult population [6].

Working to solve complex issues requires the input of all involved, and in the case of hospital to home transitions for older adults, this includes the older adults themselves [7]. In this paper we will use the term lived experience researcher (LER) rather than the more commonly used term patient partner. This was the term used by the team in this project, after discussion between the PI and LERs. The term was used to attempt to take away the power imbalance that is suggested by the term “patient” while honouring that the LERs wanted their lived experience with the research topic to be centred in a way that a term such as “public researcher” does not [8]. Engagement of LERs on a research team needs to be done thoughtfully and carefully, as they are at risk of harm because of their unique role on the team [9]. LERs often make themselves emotionally vulnerable to the team, reliving emotional or traumatic experiences for the sake of the team and project. In addition, non-LER team members may place less value and weight on lived experiences as legitimate knowledge versus formal research education. As a result, LERs can feel tokenized, gaslit, unimportant, and alienated; such an experience can compound their medical trauma [9].

To proactively address issues of potential harm in LER engagement, Shimmin and colleagues [10] argue for trauma-informed, intersectional, and reflexive engagement strategies. A trauma-informed approach aims to resist re-traumatization with the creation of physical and interpersonal settings that promote safety, choice, autonomy, collaboration, and respect [11]. Trauma results from power differentials and therefore, a trauma-informed approach seeks to avoid reproducing these power differentials. An intersectional approach acknowledges that multiple intersecting systems of oppression contribute to an individual or group’s power and privilege and aims to disrupt the invisibility of multiply disadvantaged groups [10]. This lens emphasizes the need to include voices typically ignored by mainstream society and requires active resistance of reproduction of power and inequity [10]. The concept of reflexivity has been developed both for the areas of research methodology as well as clinical health professional practice [12]. In common between research and clinical contexts is that reflexivity is a personal process of examining and critiquing the assumptions and worldviews of oneself and the contexts in which one is embedded. The goal is to challenge and change one’s own attitudes, behaviors and actions to move towards equity and justice [12, 13]. This self-analysis should include considering interpersonal power dynamics at play, and the historical and social context in which one is embedded [13].

The principal investigator (PI; CB) wanted to enact a collaborative engagement strategy, where LERs are equal partners on the research team [14] to increase the LERs potential for impact on her project on care transitions from hospital to home for older adults. The engagement needed to be carefully designed because of the potential risk of re-traumatization because of pre-existing and persistent health and academic hierarchies that would likely re-produce themselves on this team that included academic researchers, health care policy makers and managers, and clinicians. A recent scoping review identified that while power dynamics are a well-identified barrier for LER engagement, there is less understanding of how to mitigate power imbalances [15]. Further, there have been calls to document the processes of engagement, to demystify how it is done [16]. Therefore, in this comment paper, we describe the design and implementation of a trauma-informed, intersectional and reflexive LER engagement strategy that aimed to promote LER safety, inclusion and opportunity for impact during the early phase of a research program on transitions between hospital and home (i.e., the proposal development stage).

## The project

### Context and team

This work was done in an urban city in a Canadian province. The project involved the development of a research proposal with the end goal of submitting the proposal for funding. It has been identified that including people with lived experiences in all stages of the research is important [16], and thus we engaged LERs in the beginning of the work – in developing the research proposal once funding had been attained through a local seed grant for patient and public research engagement. The LER engagement was designed to be collaborative, where the LERs were equal partners in relation to decision-making on the research team, but were not leading the research activities [14]. The PI provided parameters for the project to ensure it fit within her scope of expertise; it needed to be related to care transitions from a physical health hospital setting to home and involve an older adult population. Otherwise, the PI was seeking input from the team, diverse in role, to provide more specific direction to ensure the planned project would be relevant to the multiple groups affected by the issue, and current care contexts.

The LER engagement plan for this project was designed before the COVID-19 pandemic, but implemented during the pandemic, and thus the design was adapted from in-person to online activities. LER recruitment occurred prior to the onset of the pandemic and involved: 1) promoting the project through the Manitoba Strategy for Patient-Oriented Research (SPOR) support unit; 2) connecting with community resource workers in a low-income neighbourhood; 3) emailing recruitment posters to Winnipeg senior serving organizations. The PI visited with potential LERs in-person to explain the project, the LER role, and discuss what supports were available/what supports they required to participate.

The five LERs had lived experiences either as a patient or as a caregiver of a patient who underwent a transition from hospital back to their home. Five LERs were recruited to allow for collective power in the group, while also ensuring budgetary feasibility. They were all women, aged 52 to 70. Gender diversity was difficult to achieve; one man initially recruited did not continue with the project once the pandemic started. He was replaced with an Indigenous female LER, with the PI opting to diversify the LER group by race instead of gender to include a voice situated within a group of people who have and continue to have the worst health outcomes, due to oppression, in the geographic region of the study. The LERs represented a range of household incomes and lived in neighbourhoods where the median household income range was $31,004 to $50,397 CAD, and at least three had multiple socially marginalized identities (e.g. low income and disabled). Some lived with a spouse, some with pets and others lived alone. LERs were reimbursed for meeting-related expenses and compensated for their time.

Our full research team included:

- five LERs,
- the PI (a registered occupational therapist),
- a project coordinator,
- two provincial-level policy makers in continuing care/home care,
- a front-line manager for a transitional care program,
- two clinical researchers (a physician and pharmacist),
- a social worker,
- two academic researchers (a registered nurse and a sociologist/physiotherapist),
- a researcher employed by the health system focused on home care research and evaluation.

In relation to diversity, team characteristics included:

- 13 women, two men;
- 12 White Europeans, one South East Asian and one First Nations person;
- four disabled people;
- seven people in health leadership;
- 12 people with research experience;
- 12 current or former health care providers;
- five people who went home from hospital following an unplanned admission;
- nine people who are, or who have been, a caregiver for someone after an unplanned hospital admission.

The writing of this paper was led by the academic research authors. The LER authors contributed by: offering their perspectives after completion of the project with the goal of writing this manuscript; through informal communications to the PI about their experiences on the team; and providing responses to drafts. See Appendix 1 for the GRIPP-2 patient and public involvement reporting tool which outlines the involvement of the LERs in all stages of the development of this manuscript.

### Project steps

**Fig. 1:**
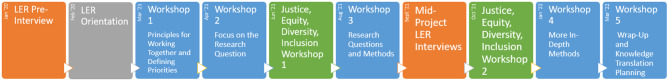
Major project steps and timeline

See Fig. 1 for the major steps and timeline of the project. We implemented commonly recognized engagement strategies recommended by public and patient researcher support and advocacy organizations [17, 18]. One of these was holding an in-person LER orientation session in January 2020 once the five LERs were recruited. Four of the five LERs attended this session while the fifth received the information via email because they were not well enough to attend. The topics of the orientation and discussion were:

1. trauma-informed approaches to working together,
2. general information about the research topic and the problem we are trying to address,
3. the role of the LERs on the research team,
4. how the LERs thought they could contribute to the team,
5. how LERs would like their participation to be supported.

All team members received a LER handbook tailored from existing materials publicly available from web-based patient researcher and local mental health support resources. Topics in the handbook were:

1. meet the team,
2. summary of the overall study process,
3. agenda for Workshop 1,
4. preparation suggestions for Workshop 1,
5. LER involvement in the research process,
6. roles for LERs,
7. Jargon Buster! (glossary of research terms),
8. free and low-cost counseling resource list.

Following a one year pause in the project after the LER orientation because of the COVID19 pandemic, the full research team collaboratively developed a study proposal in a series of seven online facilitated workshops. Table 1 provides details of the workshop objectives and engagement strategies specific to the workshops. All workshops were supported by a professional facilitator with experience managing power differentials within groups and creating and sustaining a trauma-informed, reflexive space. The project coordinator and the PI also had group facilitation roles. The original design was for six proposal development workshops, however, early in the process, the team identified equity and inclusion as an important consideration for this research topic of care transitions, where worse outcomes are associated with being a member of a socially oppressed group. A LER suggested that the team develop a Justice, Equity, Diversity and Inclusion (JEDI) statement to purposefully center JEDI in the work. Thus, the plan was adjusted to five full team planning workshops and two workshops with a sub-group to develop a JEDI Mission, Vision and Actions document (Appendix 2). The JEDI sub-group included three LERs, four researchers, and one policymaker. The subgroup shared the work generated by these two workshops with the entire team for feedback prior to finalizing the team’s JEDI statement.

**Table 1:** Workshop objectives and engagement strategies

Workshop # and Main Focus	Objective(s)	Engagement Strategies
1. Team formation and establishing direction	-Develop research team guiding principles. -Develop consensus on the research question and direction.	a. PowerPoint sent to participants in advance to support preparedness. b. Professional facilitator with no investment in the research topic and experience with legitimizing marginalized voices in a context with power differentials. c. Icebreaker activity highlighting the value of different perspectives to level power differences. d. Discussion of trauma-informed approach/team safety. e. Share group guidelines created from pre-interviews of LERs and invite changes/additions to support well-working team. f. Small group discussions in pre-determined groups to mix clinicians, policymakers and LERs into different teams. Less experienced LERs put into a group with an individual with experience working with LERs to support moderation of discussion that levels power differences and supports LER legitimacy. g. Presentation of grant-funded catalyst question and discussion of how it fits with the group’s desired priorities and outcomes of the research. h. Closing activity: What are you going to take away with you today? i. Post-workshop: Collated the main ideas from the workshop discussion and administered an online survey to confirm areas of commonality in the team re: research principles and priorities to support productive deliberation.
2. Establishing direction	-Establish consensus on research principles and priorities. -Refining the research objectives.	a. and b. above j. Icebreaker activity: “Getting to Know One Another” k. Review guidelines developed in session 1 for working together. l. Present areas of commonality from the post-workshop 1 survey. Discussion on these priorities and principles. m. Wrap-Up activity: “One thing you will do to take care of yourself today.” n. Workshop 2 non-attendees invited to complete a survey on topics/ideas that prompted discussion in Workshop 2. Survey format: audio feedback to PowerPoint slides presenting the context and question for team members.*
Justice, equity, diversity, and inclusion (JEDI) Session 1*	-Broad discussion to shape JEDI Vision and Mission.	b. and h. o. All participants share “What does EDI mean to me?” p. Review some examples of EDI Vision and Mission statements. q. Discussion on: Looking at equity, diversity and its purpose in this research project - what is the purpose of it? What are the most important aspects that we want to see?
3. Developing the plan	Discuss a proposed aim, objectives, population, and methods.	a. b. and m. c. Icebreaker activity: “Name Story” d. Review established principles and priorities for our work (Summary of Workshops 1 and 2) and “sticky points”. e. Presentation of draft aim, objectives, population, and methods and open discussion of same. f. Review of progress to date and next steps. g. Discussion of how the team wanted to be involved in the proposal writing process.
JEDI Session 2*	Review and refine JEDI statement.	a. b. k. m. r. Review draft and discussion on changes/additions.
4. Refining recruitment and data collection tools	-Review plan for research proposal submission and roles of team members. -Refine data collection strategies.	a. b. k. m. s. Icebreaker activity: “Your favourite season and something you love to do in it.” t. Sharing how the input of LERs and policymakers has influenced the project to date* u. Sharing funding target and roadmap to achieve funding application submission. v. Invite team members to review the grant components that fit best with their skills and interests. w. Discussion of data collection strategies and timing for different groups (patients, policymakers, etc.). x. Post workshop – email communication with team members to refine proposal. LERs were offered a phone feedback option.*
5. Wrap-up and feedback on working together	Evaluate how we worked together and how we want to work together going forward.	a. b. k. m. y. Reflection questions: -What is a) one personal thing you feel you have been able to achieve during this project; and b) one thing achieved as a group during this project? -Looking at personal/group successes - what do we have to ensure success moving forward as a group? -Considering what we have achieved and learned is there anything that we should be sharing more widely right now? -Are there other perspectives/organizations that we need to include moving forward? z. Sharing next steps/future timeline re: grant submission.

*Elements that were added to the design during the project based on feedback mechanisms and needs of LERs

### The trauma-informed, intersectional and reflexive LER engagement strategies

In addition to implementing commonly recognized engagement processes, this project had a special focus on taking a trauma-informed, intersectional and reflexive approach to the LER engagement [10]. The main way the approach was different was that the process of relationship-building and maintenance was prioritized over the desired outcome of a completed research proposal. This was to ensure that the goal of the outcome did not supersede the well-being of the LERs in working to reach that outcome. Primary foci were in reducing embedded health and research hierarchies and ensuring psychological safety and legitimacy of the LERs. While there was great effort to ensure project progress, the project moved thoughtfully, and when needed, slowly, to ensure the continued preservation of team member safety and feelings of inclusion.

All team members were invited to engage in reflexive thinking throughout the entirety of the project. A few examples include:

- LER recruitment: the PI and project coordinator considered how their positionality as White women may influence recruitment and reflect on how to mitigate potential bias;
- LERs shared how they could be best supported in the project based on their past experiences and specific needs;
- team members considered their positionality in territorial acknowledgments that opened each workshop;
- team members discussed how they could create a safe space despite group diversity through the development of group guidelines;
- team members both present and not present at meetings were acknowledged so the team could consider which perspectives were present and missing at each workshop;
- team members learned how their perspectives were different, strengthened understanding of commonalities, and strengthened relationships through getting to know each other through warm-up and wrap-up activities. One example was having everyone tell the story of the origin of their first name as an opening activity; another activity that brought together commonalities was a closing activity where everyone shared how they would engage in self-care that evening.

The workshop facilitators used prompting questions from Shimmin and colleagues [10] to consider design elements that would be supportive to the LERs. One example of this was having small group discussions in pre-determined groups to mix clinicians, policymakers and LERs so that less experienced LERs were always in a group with another team member with experience working with LERs.

Consistent with a trauma-informed approach that emphasizes autonomy and choice, LERs were able to tailor their participation to their needs and preferences. The PI and project coordinator connected with LERs between workshops using their preferred communication strategies to prepare them for, or request feedback on, workshop topics.

### Feedback mechanisms

To support continual evaluation and adjustments to support safety and inclusion of the LERs, feedback mechanisms were incorporated. Use of feedback mechanisms is key to a reflexive approach, to allow for evaluation and adjustment of the LER engagement strategy throughout the project [10]; such reflexivity and iterative strengthening of team processes may be key to team satisfaction and productivity [19]. Thus, monitoring the group process is recommended to identify and address problems in collaborations, relationships, strategies, and avoid attrition [19]. We received ethical approval for the inclusion of these feedback mechanisms in the project, and all team members (including LERs) provided written consent for the gathering and analysis of the feedback data.

The primary feedback mechanisms were interviews with the LERs and analysis of group dynamics in workshops. Two interviews were conducted before project completion with each of the five LERs: the first was conducted prior to the LER orientation and the second was conducted mid-project. The interview guide development was informed by the qualitative questions of The Public and Patient Engagement Evaluation Tool (PPEET) designed by McMaster University to help organizations assess their engagement activities [20], as well as the Shimmin and colleagues paper [10]. We categorized the data using LER engagement indicators developed by Alberta Health Services [17], including representativeness, inclusivity, transparency, decision-making, task definition, comfort, satisfaction, fairness, and communication with the primary goal of planning/adjusting subsequent engagement to meet LER needs.

The second feedback mechanism was analysis of group dynamics from transcripts of workshop recordings. Between workshops, one researcher (PT) analyzed the within-workshop dynamics, then shared feedback with the workshop facilitators (PI and professional facilitator). PT applied core principles of conversation analysis to do this. Conversation analysis understands talking is action [21]. Through talk, we do actions like expressing agreement or disagreement, inviting clarification, asking questions, issuing commands, and more. Both the words said and how they are said matters; pauses, volume, interruptions and more propose a way to understand an utterance. But in conversation analysis, the meaning of any given utterance is not given; instead, as others respond to it, a co-constructed meaning is developed within the ongoing conversation [22]. A comment, for example, can spark hesitation, disagreement, or enthusiastic agreement.

PT focused on group dynamics by considering the conversation turn-by-turn in sequences on a topic, rather than focusing on particular statements in isolation [23, 24]. PT did not conduct a full conversation analysis; to do so requires a detailed, rigorous transcription method be used that includes phonetic spelling, overtalk and interruptions, intonation markers, precise timing of pauses, and more. Instead, PT applied the principles, looking for sequences where talk that proceeded smoothly and others where there were difficulties [21]. She reviewed what created interactional difficulties, and how difficulties were resolved within the workshops. PT offered possible interpretations to the facilitators, who considered and discussed this feedback in relation to their own written reflections that included impressions of the workshop dynamics. These reflexive discussions provided additional information to consider in the preparatory and debrief meetings for each workshop. An example was discussing and considering if the PI was responding in a consistent way to LERs and academic researchers when they contributed information that seemed to her to be tangential to the project goal.

Additional reflexivity strategies were that the PI regularly reflected in writing on how she was experiencing the dual role of being the PI and facilitating the group process and noted personal areas of tension. Further, the project coordinator and PI both reviewed feedback from all team members that were received in all forms, both solicited and volunteered from team members.

## Reflections

The use of Shimmin and colleagues’ [10] approach supported the academic researchers in being aware of, and attempting to reduce hierarchies on the team, using relational approaches. It also prompted the researchers to invite the LERs to also engage in reflexive thought, which helped to gain a deeper understanding of their needs. The approach resulted in the emphasis of relationship-building and maintenance over a speedy outcome and moving towards a de-hierarchical approach to knowledge building. Of course, the dismantling of long-embedded academic and health hierarchies cannot be accomplished in one project. However, the LER authors of this commentary with previous LER experience found that this project felt different than other projects they had engaged in as LERs and appreciated how they were valued as equal team members. The LERs think that the trauma-informed, intersectional and reflexive approach should be considered for wider and explicit application in health care settings, noting how the feeling of empowerment in this project contrasted with many of their health care experiences. A trauma-informed approach would emphasize physical, psychological, and emotional safety and enhance wellbeing by empowering individuals to define their needs and goals and make choices about their care and services [25].

The academic authors of this commentary also noticed that this relational approach changed the way we thought about relationship-building on a research team. Primarily, this experience pointed out how important it was to be very deliberate with the relationship-building strategies, and how important it was to be simultaneously working on group relationship-building for the entire research team as well as with individual relationship-building with the LERs. Every interaction and non-interaction provided opportunities to either perpetuate or disrupt social hierarchy and power imbalances. The academic research authors have experience as health care clinicians, and so a relational empathic approach to promote health and healing is a common value that we strive to enact [26]. However, using the feedback mechanisms allowed us to develop deeper understanding of how power imbalances were affecting LER engagement, and sometimes prompted transformative moments of unearthing personal bias which allowed for it to be unlearned and addressed. Here, we share examples of how the feedback mechanisms in combination with reflexivity supported the academic researchers to support engagement.

The first example highlights how inviting and respecting the LERs own knowledge about their participation needs supported their engagement. The pre-interviews supported learning about the LERs by having an interviewer with clinical counseling experience who was able to enact trauma-informed principles, use evocative empathy to support the LERs feeling heard, and who had experience inviting and making room for personal stories. The interviewer validated the experiential knowledge of the LERs and the importance of their contributions. This approach requires special training in active listening and in guiding the interview, rather than leading it [27]. With this approach, the LERs were able to be very specific in what would support them during the research workshops. The PI and project coordinator continued to follow-up with the participants between workshops to continue to evaluate how their participation could be supported.

The PI had thought that since all the participants had agreed to participate in the project, they would be comfortable with group meetings, however this was not the case. The LERs indicated varying needs in the in the pre-interviews in relation to privacy, information processing and knowledge levels. Suggestions they had for support included ensuring that discussion was facilitated so that they were not spoken over or dismissed by other team members, not being ‘put on the spot’ in workshops, being given direction on what information they should attend to most due to time constraints due to caregiving responsibilities, and when we transitioned to online, attending with camera off, or participating only by reviewing materials rather than attending meetings. This information gleaned in the pre-interviews resulted in a change to the LER engagement plan so that LER participation was individualized. While in retrospect this need to individualize engagement for a group of people who had lived experience in the health care system as patients or as caregivers for older adults is not surprising, it was eye-opening in the moment as the plan had been to take a universal design approach to LER engagement. However, the more individualized approach was more in alignment with the intention of a trauma-informed approach, where provision of autonomy and choice can empower people to engage in ways that maintain their sense of control and safety [25]. It was not expected that the pre-interviews would be so critical to the LER engagement planning as they were initially primarily included for evaluation of the impact of the LERs on the project. However, it is now clear that the relational and reflective interview approach by someone with strong training was important for setting the tone for this project with the LERs. In designing engagement strategies, the plan needs to consider that every contact matters.

A second example of how the feedback mechanism in combination with reflexivity supported engagement efforts was that the PI and PC learned mid-project that some of the LERs were not feeling involved in the project development. The mid-project interview indicated that some LERs felt like there were groups splitting off to do different things they were not aware of, and that there was a lack of communication. This prompted the PI to realize that she had unintentionally been taking a paternalistic approach of “protecting” the LERs and gatekeeping information, such as finalizing the details of the theoretical construct and methods, assuming the LERs would have less of an understanding and interest of the methodological details. While this was rooted in good intentions to prevent overwhelming the LERs with unnecessary information and jargon, she realized she was perpetuating practices that reinforced hierarchies of knowledge and information. While literature cites challenges with having methodological discussion with LERs as a main challenge of meaningful engagement [8], this feedback was a reminder to let the LERs choose their path of engagement rather than making decisions for them under the guise of protecting them.

A third example of important reflection that occurred that supported engagement near the end of the project was the feedback from the LERs that they felt an urgency for influencing change in an immediate way. The PI communicated to the LERs that research is a slow process to support realistic expectations. However, the LERs pointed out that for an important health issue, it was crucial to take immediate action, and not just be content with working towards advocacy in the future. Thus, two academic researchers and three LERs wrote an editorial for the local newspaper together to highlight the issue in the community using research and knowledge developed by other researchers. This challenge from the LERs to have immediate impact disrupted stories the PI had created about what the “right order” of research activities, and about “staying in one’s own lane” and focusing on one’s own research for knowledge translation. While the PI likes to think that she does not value the research structure of “independent researchers”, and longs for a more collective policy-informing approach to research, she was not practicing in this way.

These three examples have highlighted that the process of formally eliciting feedback allowed us to course correct to maintain relationships, support continued LER engagement and to provide an opportunity to critically examine our practices as academic researchers. While many engagement enablers have been identified in the literature, to our knowledge, our commentary is one of the first to describe the use of reflexivity in an engagement project. We found one other paper that highlights the importance of reflective practice, but not reflexivity, which is conceptually different. Reflexivity is important for considering power structures and how they are being enacted [28]. In order to be able to be reflexive about the power dynamics at play with the research team as a whole, group process analysis was a critical skill for the facilitators of the workshops [29]. The PI had group process skills due to her professional background as an occupational therapist. Despite this, the analysis and facilitation of the group process was challenging because of the PIs investment in developing a strong research product and the complex social dynamics at play. As such, the use of a professional facilitator was essential, as was having the project coordinator, whose role was to support logistics during the workshops.

Further, the group dynamics analysis by another party not involved in facilitation engagement helped to support reflexivity and confirm or oppose impressions during workshop debriefs and planning. The approach we took of recording and reflecting on the workshops may be a powerful learning tool for researchers new to facilitating group process. Additionally, researchers new to group process need to ensure that they have strong support from others who are experienced in group process. Careful facilitation is required to genuinely achieve an engagement level to “collaborate”, where the intention is for the LERs to be an equal partner on the research team [14]. Through careful facilitation and offering a variety of options for providing input, the LERs were supported in speaking up despite power imbalances [8].

Going into this project, it was accepted that trade-offs between efficiency and using the relational approach would be needed, as impact on efficiency is a well-known challenge with LER engagement [8]. The PI prioritized relationship-building processes over perceived efficiency, trusting that taking the time needed to form effective working relationships would enhance the research product. While there is encouragement in the engagement literature to use collaborative approaches to bring power to the LERs, the researchers also need to have specific skills and the resources to do this engagement well. The risk of continuing to harm and perpetuate power imbalances by inviting LERs to participate without attending to their needs or ensuring their voices are heard is too high.

### Limitations

The LERs were not a part of the initial planning of the design of the engagement strategy, however, they had input in to how they were involved and in priority setting. Their early participation in the project allowed for a shift in the research plan from what the PI originally proposed. This article provides a detailed description of our engagement method, along with reflections from all authors of this paper, and thus the reader is invited to engage a critical lens when considering our reflections. While the academic researchers led the writing of the article, LERs were involved in the discussions that led to the article and were invited to engage in multiple rounds of feedback in whatever way they chose, whether that be editing the article or providing comments. The publishing process for this paper involved multiple rounds of revisions and long waits between revisions for reviews that strengthened the paper but resulted in fewer comments from LERs as the process continued. However, the LERs continually reinforced that the main messages that they wanted to share were captured in the article. The LERs main interest for publication was in relation to the team’s research topic. The LERs were involved in the writing of an Opinion Editorial for a newspaper to ensure that the public was informed of issues important to them related to the research topic as a part of this project.

This paper does not address barriers and facilitators of engagement at an institutional level, other than identifying institutional funding as a support for our LER engagement. Further, this paper does not report on empirical evaluation of the engagement. An evaluation of the impact and outcome of the LER engagement strategy was completed which we will share in a future publication.

## Conclusion

This paper shares the process of collaboratively developing a research proposal using a power-focused framework with a team with multiple LERs to help to address a gap in the literature: what are the specific ways that researchers can support the reduction of power hierarchies when engaging LERs? While there are resources to support researchers in learning about the types of engagement they could use, there is little guidance on how to decide which one to use [30, 31]. Therefore, we offer these reflective questions when a PI is considering engagement at the level of “collaborate” with systemically marginalized groups:

- Am I willing/able to pause the progress of the research if that is necessary to ensure that LER needs and voices are attended to?
- Am I committed to being open to, and being uncomfortable as I begin to learn the ways that power imbalances manifest in my project?
- Do I have at least one, and preferably more, people on the research team with experience in trauma-informed approaches, group process and active listening skills?
- Do I have someone on the team that will have enough time and skills to work one-on- one with any LERs who are needing accommodations, support, information and/or opportunities to share their input in a variety of modalities?
- Do I have a facilitator to support meetings who has experience in trauma-informed care and facilitating groups in which power differentials exist? This should not be the PI, even if they have this training.
- Is this a research topic that requires different perspectives in all aspects of the research cycle? For example, for the topic of care transitions, research advancement has stalled and thus requires new approaches and perspectives.
- What power-focused model will I use to explore and overcome researcher-LER power imbalances? A scoping review by Greenhalgh and colleagues identified 13 published power-focused frameworks that can guide researchers [32].

We hope that our tangible examples of the benefits and drawbacks of using a power-focused framework within a project designed to be collaborative will support researchers in their decision-making about what level and extent of engagement makes sense for their research topic and their skills and resources. This careful reflection and planning will support improved collaboration between all research team members and reduction of harm to LERs.

## Electronic supplementary material

Below is the link to the electronic supplementary material.

Supplementary Material 1

Supplementary Material 2

## Data Availability

The materials on which this paper are based are not publicly available but readers interested in more information about the project are invited to contact the corresponding author on reasonable request.

## Abbreviations

GRIPP-2: Guidance for Reporting Involvement of Patients and the Public Version 2
LER: Lived experience researcher
