# Supplementary material for: Disrupting power hierarchies: applying a trauma- informed, intersectional, reflexive engagement strategy
Source: Res Involv Engagem. 2026 Jul 1;12:104. doi: 10.1186/s40900-026-00930-4 (PMC13321561; doi:10.1186/s40900-026-00930-4)
Supplement: Supplementary file 2 — Supplementary Material 2 [file 40900_2026_930_MOESM2_ESM.docx]

***Appendix 2***

**Justice, Equity, Vision and Mission Statement for Program of Research:**

**Stimulating a Shift to Co-Designed Care for Transitions from Hospital to Home**

*This JEDI statement is a North Star for our project team and project. JEDI will drive our activities and decisions, including determining and allocating resources. When we are at a cross-roads, need to make a major decision, or are in a state of uncertainty, we will to return to this statement for guidance. Our JEDI statement is a fluid document.*

**Vision**

- That all Manitobans transitioning between hospital and community participate in co-designed care* regardless of language, race, ethnicity, sexual orientation, gender, social and economic situation, education, health literacy, digital access, religion, ability, or geographical location.

*co-designed care: care that meaningfully centres and facilitates participation of the person and their circle of care to the extent that the person desires, and in a way that meets the person’s needs. This definition will evolve through the research project.

**Mission**

- To embody an inclusive research process guided by our vision and values. This may require dismantling barriers in order to include previously unheard voices in all stages of the research process.

**Actions**

- We will use a relational approach. This means “prioritizing our relationships with community partners and community members over settler-colonial and institutional expectations of professionalism, efficiency, and success.” (Ferland, Chen & Villagrán Becerra, nd)
- We will strive to be anti-racist, anti-oppressive and anti-colonial in our work. We will seek to identify when colonial and/or institutional structures are impeding JEDI and advocate for change to these structures. We will use humility to accept that our own perspectives are limited, to value all expertise equally, to recognize the range of expertise from lived experience to empirical research, and to embrace the importance of all perspectives.
- We will be flexible in our processes and approaches in order to integrate new perspectives, knowledge and needs over time.
- We will not make assumptions about individuals and their needs. E.g. social supports, access to services, people’s context/needs/wishes.
- We will mitigate the trauma inherent to colonized health care and research practices by using a trauma-informed approach.
- We will make our work accessible to all people involved in the project, and to all those it will potentially affect. This includes recruiting and supporting less heard voices and addressing individual needs for participation related to needed accommodations and/or practical support.
- We will use an intersectionality approach where we recognize that all the different identities of an individual contribute to their personal experience. This will ensure that our team, participants, and results adequately address the richness and complexity of human experiences.
- We will carefully consider the need for resources, and the allocation of resources in order to support growth of the team in being able to do JEDI work, as well as ensure those structurally disadvantaged can engage in our work.

*Actions Specific to Research Team Culture*

- - Disrupting discrimination is not an activity that is isolated to this research project – team members will strive for JEDI lens in their work and play.
  - We will engage in continuing education related to increasing our capacity for JEDI work.
  - We will strive to create a safe space where team members can challenge each other’s thoughts in supportive and constructive ways.
  - We will acknowledge and accept that everyone is in a different place in learning about, and addressing their own personal biases. We will support all team members in advancing in their learning journey nonjudgmentally, as long as no one’s safety is being compromised.
  - We will commit resources for the research staff team to engage in continuing education and personal growth activities that will enhance their knowledge and skills in disrupting discrimination.
  - We will acknowledge and value the intersectionality of our research team members. Everyone is a multiplicity of identities that makes their personal identity and experience unique.

**References**

Ferland, N., Chen, A. & Villagrán Becerra, G. (nd). Working in Good Ways: Relational Assessment Guide. University of Manitoba. https://umanitoba.ca/sites/default/files/2021-05/relational-assessment-guide.pdf
